# Transcranial Direct Current Stimulation and Mirror Therapy for Neuropathic Pain After Brachial Plexus Avulsion: A Randomized, Double-Blind, Controlled Pilot Study

**DOI:** 10.3389/fneur.2020.568261

**Published:** 2020-12-11

**Authors:** Clarice Martins Ferreira, Carolina Dias de Carvalho, Ruth Gomes, Erickson Duarte Bonifácio de Assis, Suellen Marinho Andrade

**Affiliations:** Neuroscience and Aging Laboratory, Federal University of Paraíba, João Pessoa, Brazil

**Keywords:** brain stimulation, mirror therapy, chronic pain, brachial plexopathy, peripheral nervous system diseases

## Abstract

**Introduction:** Although transcranial direct current stimulation (tDCS) and mirror therapy (MT) have benefits in combating chronic pain, there is still no evidence of the effects of the simultaneous application of these techniques in patients with neuropathic pain. This study aims to assess the efficacy of tDCS paired with MT in neuropathic pain after brachial plexus injury.

**Methods:** In a sham controlled, double-blind, parallel-group design, 16 patients were randomized to receive active or sham tDCS administered during mirror therapy. Each patient received 12 treatment sessions, 30 min each, during a period of 4 weeks over M1 contralateral to the side of the injury. Outcome variables were evaluated at baseline and post-treatment using the McGill questionnaire, Brief Pain Inventory, and Medical Outcomes Study 36–Item Short-Form Health Survey. Long-term effects of treatment were evaluated at a 3-month follow-up.

**Results:** An improvement in pain relief and quality of life were observed in both groups (*p* ≤ 0.05). However, active tDCS and mirror therapy resulted in greater improvements after the endpoint (*p* ≤ 0.02). No statistically significant differences in the outcome measures were identified among the groups at follow-up (*p* ≥ 0.12). A significant relationship was found between baseline pain intensity and outcome measures (*p* ≤ 0.04). Moreover, the results showed that state anxiety is closely linked to post-treatment pain relief (*p* ≤ 0.05).

**Conclusion:** Active tDCS combined with mirror therapy has a short-term effect of pain relief, however, levels of pain and anxiety at the baseline should be considered.

**Clinical Trial Registration:** www.ClinicalTrials.gov, identifier NCT04385030.

## Introduction

Brachial plexus injury-related neuropathic pain is a complex chronic complication with a broad variety of structural and functional brain changes involved (1–3). Patients with traumatic lesions of the brachial plexus report compromised quality of life associated with pain, functional limitations, and other symptoms, including emotional consequences (4, 5).

Transcranial Direct Current Stimulation (tDCS) is increasingly used to treat refractory chronic pain (6–8). It is a safe and non-invasive therapeutic method that changes cortex excitability according to the stimulation polarity, producing long-lasting effects (9). In addition, previous studies suggested the use of tDCS as an approach to enhance or “boost” physical therapy (10, 11). A commonly used form of rehabilitation is mirror therapy (MT), which involves a mirror being placed in a position that allows the patient to view a reflection of a body part (12). A growing body of evidence shows that the mirror creates visual feedback of what appears to be movement of the affected limb and this visual input might reduce the activity of systems that perceive protopathic pain (13–17). This intervention has also been demonstrated to be significantly beneficial for cortical reorganization (18).

It has been suggested that combining tDCS with a rehabilitative protocol for pain can foster the effects of single treatments (19). Given that most studies were conducted in other populations, such as Parkinson's Disease (20) and stroke (21, 22), remarkably, no studies have explored the synergistic effects of these techniques in patients after brachial plexopathy. In this work, we sought to investigate whether tDCS and MT may induce pain relief and improvement in the quality of life in neuropathic pain due to traumatic brachial plexus injury. We hypothesized that active tDCS combined with MT would lead to analgesic effects and better quality of life. We also explored whether baseline clinical and demographic characteristics were predictors of response.

## Methods

### Participants

Thirty participants with brachial plexus lesions were recruited from public health units from September of 2019 to December of 2019. This study was conducted in accordance with the principles of the Helsinki Declaration. All subjects gave informed consent in a written form, according to the protocol approved by the Institutional Ethics Committee.

Participants were assessed for eligibility based on the following inclusion criteria: age over 18 years; chronic neuropathic pain for at least 3 months following traumatic plexus injury; a pain intensity of at least 4 out of 10 in the numerical rating scale; stable pharmacological treatment for at least 1 month prior to the study and throughout the study. The diagnosis of a traumatic brachial plexus lesion was based on clinical criteria based on motor, sensory, and autonomic deficits (23–25) and corroborated by neurophysiological criteria (26). We also applied DN4 Questionnaire (27) to confirm the presence of neuropathic pain (28), according to the American Academy of Neurology recommendations (26). Exclusion criteria were; severe pain of another origin, such as musculoskeletal pain; alcohol or substance abuse; associated peripheral or central nervous system diseases, and contraindications for non-invasive cerebral stimulation.

### Study Design

A randomized, controlled pilot trial was conducted, wherein participants admitted consecutively were assigned to 1 of 2 treatment groups (1:1 ratio): active tDCS plus MT or sham tDCS plus MT. Registration and randomization were centralized via a web-based system https://www.random.org/. Group allocations were sealed in opaque envelopes, which were kept by a third-party researcher. The same staff member, who was blind to the treatment interventions, performed all clinical evaluations. An independent researcher, who applied the interventions, remained blind to the findings of the clinical evaluation. Patients were masked to treatment allocation and the hypotheses of the study.

This paper has been presented according to the CONSORT guidelines (Supplementary Table 1) (29). Due to the exploratory nature of this pilot study, a sample size calculation was not performed.

### Assessments and Outcomes

In the baseline visit, we performed the structured questionnaire including demographic data, injury characteristics, affected side, pain severity, symptoms of depression, and anxiety. Pain severity was evaluated with a Visual Analog Scale (30). The Beck Depression Inventory (BDI) was used to assess the severity of depression (31). The State-Trait Anxiety Inventory was used to measure two different components of anxiety, state, and trait (32).

The primary and secondary outcomes were applied at baseline and post-treatment. The score change in McGill Questionnaire (MPQ) was the primary endpoint of our study. The McGill Questionnaire is a multidimensional instrument that evaluates various aspects of pain: sensory, affective, evaluative, and miscellaneous. The quantitative data is summed to form the Pain Rating Index (PRI) with a score rating from 0 to 78, lower scores reflecting more desirable conditions (33).

The secondary outcome measures were pain related to daily activities and quality of life. The Brief Pain Inventory (BPI) was designed to measure both the intensity of pain and the interference of pain on daily activities. The four severity items and the seven interference items can also each be averaged to form the Pain Severity Index and the Pain Interference Index (34). Quality of life was measured according to the Medical Outcomes 36-item Short-Form Health Survey (SF-36), a self-reported instrument consisting of 36 items divided into eight dimensions of health-related quality of life. The scores range between 0 and 100, with higher scores representing better health (35). To assess safety, we used a tDCS questionnaire based on previously reported adverse events (36).

### Interventions

#### tDCS

**Figure 1:**
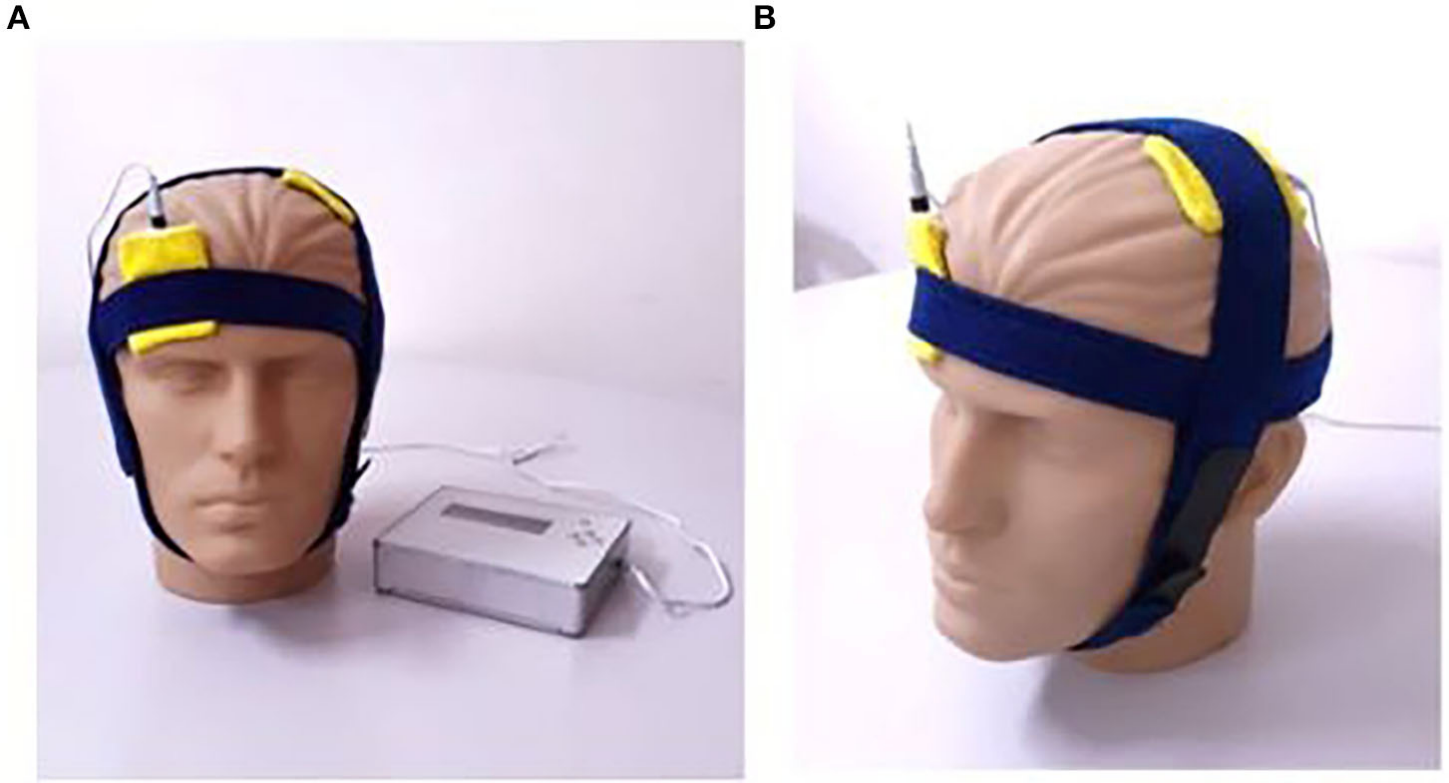
**(A)** TransCranial Technologies neurostimulator and experimental configuration. **(B)** Stimulation over the M1 contralateral to the affected upper limb.

All of the patients received tDCS stimulation, starting at the beginning of MT, over M1 contralateral to the side of injury (Figure 1). Each participant received 12 treatment sessions, 30 min each, during a period of 4 weeks. We applied a constant current of 2 mA (current density = 0.80 A/m2) delivered by a battery-powered stimulator (TransCranial Technologies, Hong Kong, China), using two 25 cm^2^ surface sponge electrodes soaked in a saline solution (0.9% sodium chloride). The anodal electrode was placed over C3 or C4 (according to the International System 10–20) and the reference electrode was fixed on the contralateral supraorbital area. Sham tDCS was applied with the same configuration, but the current lasted only 30 s.

#### Mirror Therapy

**Figure 2:**
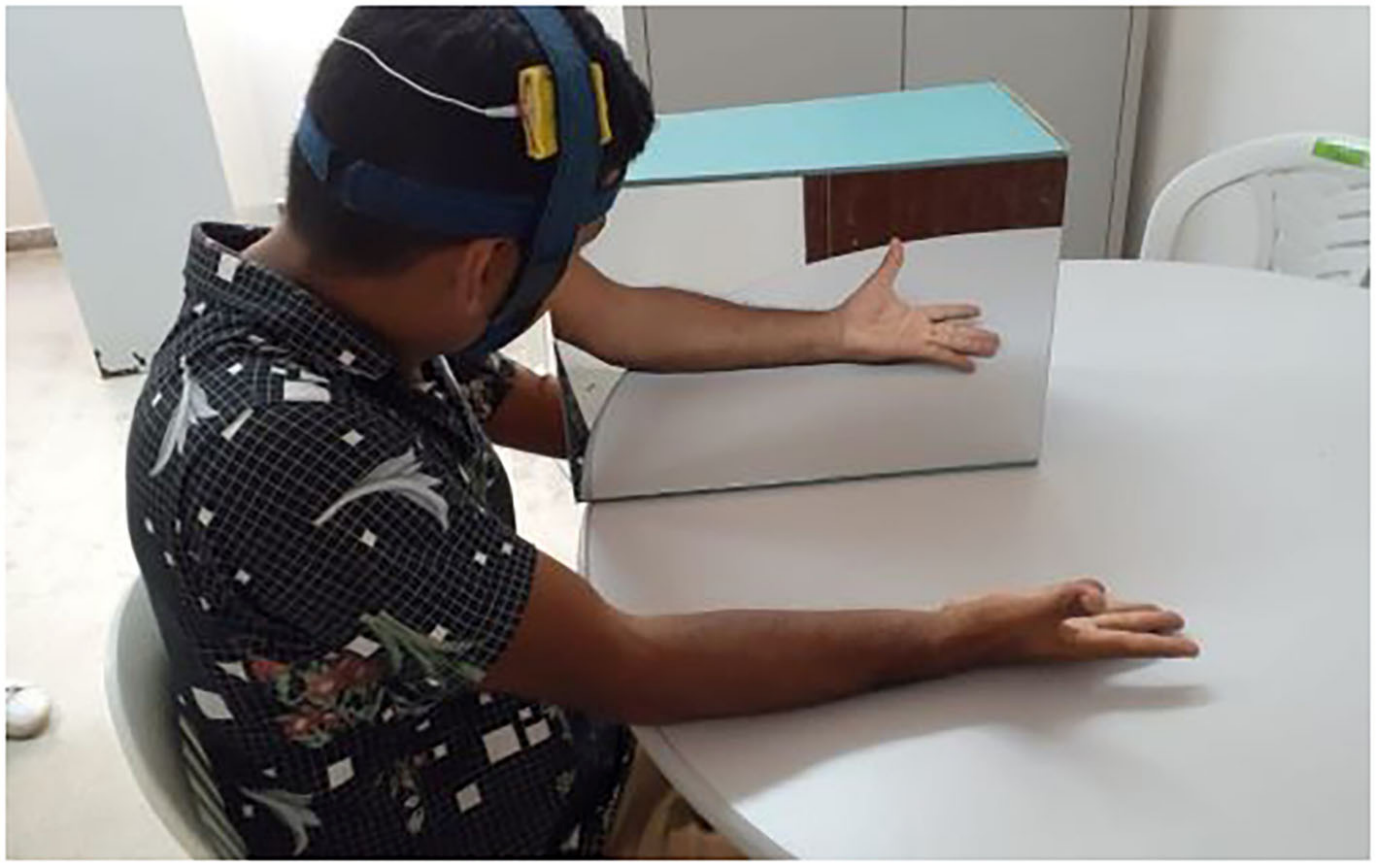
Patient performing the intervention with mirror therapy.

The intervention was delivered 3 days per week and all sessions were performed at the same time of the day, in the same quiet room. Participants were seated close to a table on which a mirror (30 × 45 cm) was placed vertically (Figure 2). The involved hand was placed behind the mirror and the non-involved hand in front of the mirror. The patients observed the reflection of their unaffected upper limb while performing the following movements (bilateral arm training): wrist flexion, extension, circumduction, radial and ulnar deviation, fisting, releasing, abduction, and adduction of all fingers. Activities were graded during the following weeks of training (37, 38).

### Statistical Analysis

An intention-to-treat analysis was applied during the study. Comparisons of the baseline characteristics between groups were analyzed using the chi-squared and Fisher's test. K-related samples Friedman test was applied to study the effect of tDCS on such variables over time, with *post-hoc* Dunn-Sidak adjustment. Mann–Whitney *U*-test was run for pairwise comparison. Further, we determined the overall effect size using Kendall's coefficient of concordance (within-group change) and Cliff's delta test (between-group change). All tests were 2-tailed and differences were considered statistically significant at *p* < 0.05.

Due to many factors that may influence pain level and quality of life after treatment, we adjusted the value in a linear regression model with the stepwise method, which tested the influence of age, duration of illness, pain severity (VAS score), and baseline depressive symptoms (BDI score). We also included anxiety (using STAI-S) as a covariate because pain and quality of life may be affected by state anxiety level. We tested single effects for treatment (active tDCS plus MT and sham tDCS plus MT) and anxiety levels, and interaction between treatment and pain severity at baseline, followed by Bonferroni correction and univariate linear regression analyses where significant main effects were found. To avoid baseline differences, we used deltas (Δ) based on the mean difference's calculation [(post-test–pre-test)/pre-test] for these analyses. All statistics were conducted using Statistical Package for Social Sciences v21 (IBM, Armonk, NY).

## Results

**Figure 3:**
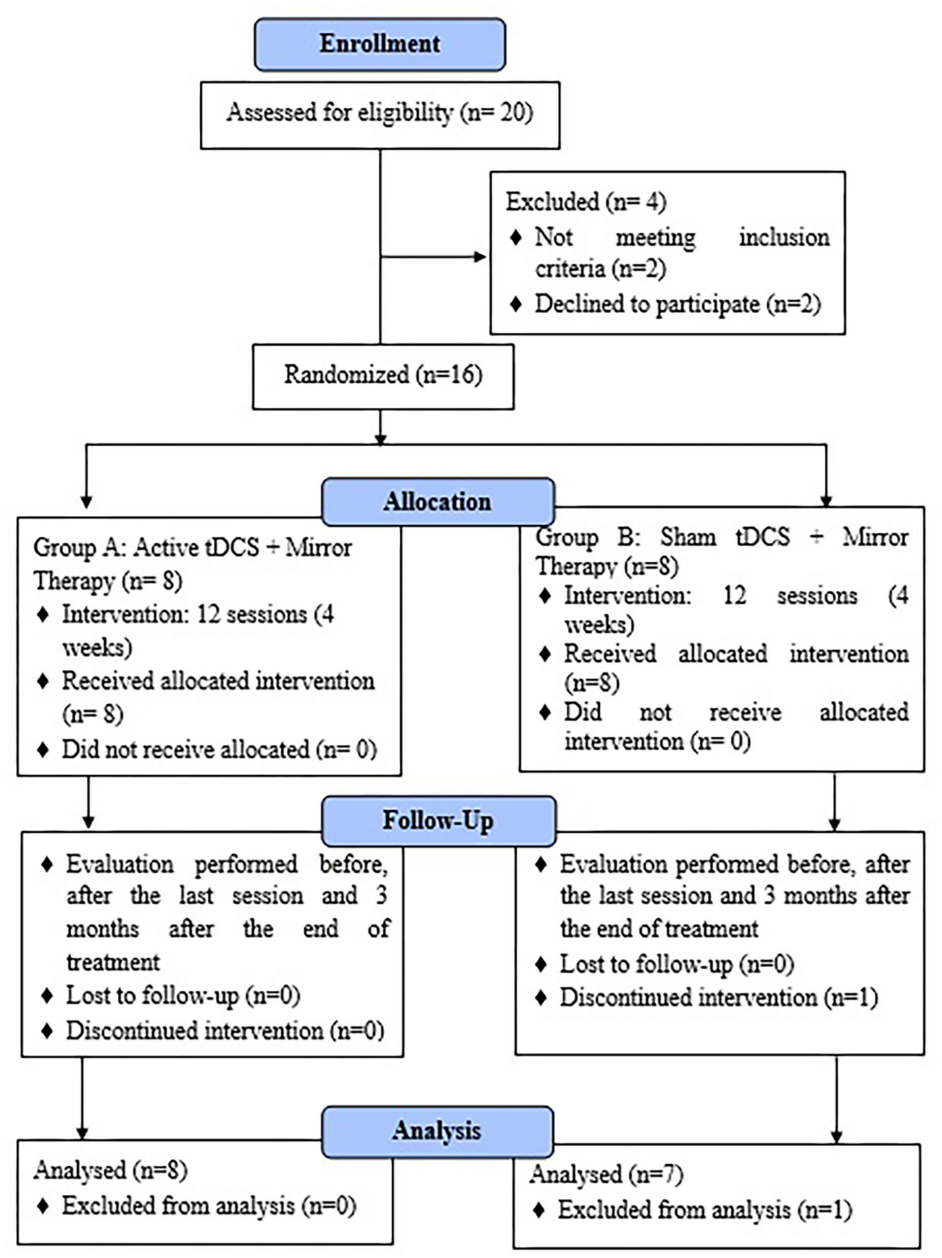
Flowchart of the participants passing through the study.

Sixteen patients were randomly assigned to one of two study groups (active tDCS + MT and sham tDCS + MT). Only one patient failed to complete the entire study. Both active intervention and sham were well-tolerated. Two patients in the active treatment group described a transient mild tingling or a slight itching sensation associated with the onset of stimulation (Figure 3).

### Demographic and Clinical Characteristics

Table 1 presents the baseline demographic and clinical characteristics of the participants. The demographic characteristics were not significantly different between the two groups. Also, clinical variables (illness duration, affected side, and scores on BDI, STAI, and VAS) were statistically similar between the active and sham treatment groups.

**Table 1:** Demographic and clinical data at baseline assessment.

**Variable**	**Group sham**	**Group active**	***p*-value**
Age, years (Mean ± SD)	36 (11.2)	39.63 (7.56)	0.37
Gender, *n* (Male/Female)	7/1	8/0	0.18
Injury time, years (Mean ± SD)	5.41 (1.16)	4.95 (1.67)	0.62
Affected side, *n* (Right/Left)	4/4	4/4	0.91
Etiology, *n*			
Motorcycle accident	6	7	0.82
Fire gun	2	1	0.88
BDI (Mean ± SD)	17.01 (7.07)	18.63 (8.60)	0.49
STAI–Trait (Mean ± SD)	48.02 (8.31)	48.25 (9.15)	0.52
STAI–State (Mean ± SD)	45.20 (10.06)	46.88 (7.24)	0.68
VAS	8.06 (1.98)	8.13 (1.36)	0.47
DN4	7.60 (1.14)	7.33 (1.15)	0.76

*BDI, Beck Depression Inventory. STAI, State-Trait Anxiety Inventory. VAS, Visual Analog Scale. DN4 = Douleur Neuropathique 4 Questions*.

### Primary Outcome

**Figure 4:**
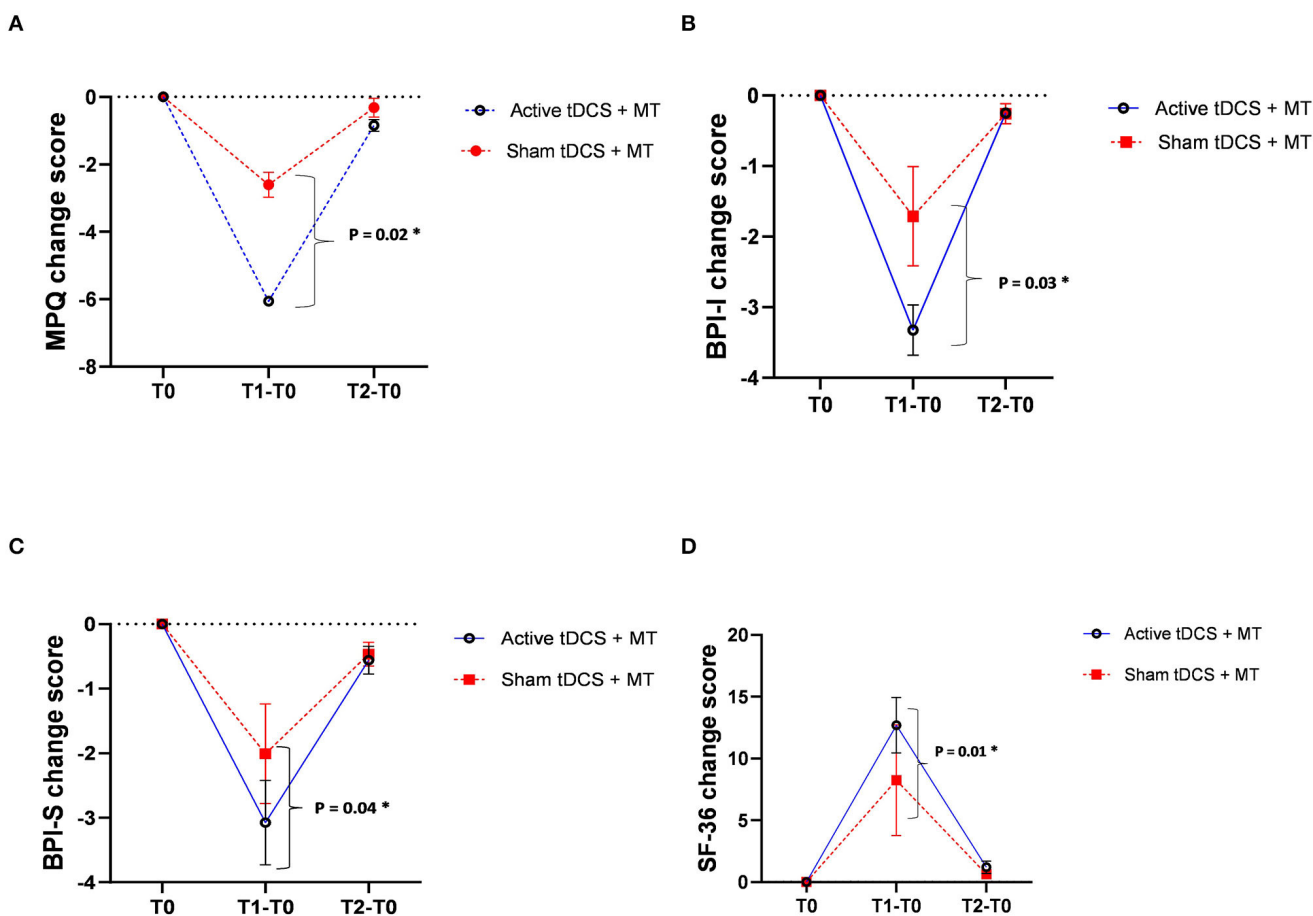
**(A)** Changes in McGill questionnaire (multidimensional pain); **(B)** Brief Pain Inventory- I (pain interference); **(C)** Brief Pain Inventory- S (pain severity); **(D)** Medical Outcomes 36-item short-form health survey questionnaire–SF-36 (quality of life) after active transcranial Direct Current Stimulation (tDCS) plus mirror therapy (continuous line) or sham tDCS plus mirror therapy (dotted line). Error bars indicate standard error of the mean (SEM). *p < 0.05.

The pain thresholds obtained from the MPQ points to a significant difference between groups, which followed the same trend: a reduced perception of pain after treatment when compared to the baseline. However, there was an apparent benefit to the active tDCS+MT group over the sham tDCS+MT group at the endpoint. The perception of pain relief was not maintained after 3-month for both groups (Table 2). The change in the level of pain from baseline to final follow-up is shown in Figure 4.

**Table 2:** Comparison of the outcome measures by groups over time points.

	**Baseline (T0)**	**Endpoint (T1)**	**Follow up (T2)**	**Within-group**			
	**Median (IQR)**	**Median (IQR)**	**Median (IQR)**	***p*-value (T1 vs. T2)**	***p*-value (T2 vs. T3)**	***p*-value (T1 vs. T3)**	**W**
**MPQ**							
Active tDCS + MT	14.04 (12–16)	8.02(6–9)	12.75 (12–16)	0.01^*^	0.04^*^	0.438	0.17
Sham tDCS + MT	13.11 (11–16)	10.37(9–12)	12.46 (10–15)	0.01^*^	0.03^*^	0.719	0.10
Between-group effects *P*-value (δ)	0.44 (0.01)	0.02^*^(0.68)	0.18 (0.03)				
**BPI interference**							
Active tDCS + MT	7.49 (7–9)	5.13(5–7)	7.14 (7–9)	0.01^*^	0.03^*^	0.63	0.41
Sham tDCS + MT	8.05 (7–9)	6.17(6–8)	7.96 (7–9)	0.03^*^	0.01^*^	0.24	0.37
Between-group effects *P*-value (δ)	0.67 (0.04)	0.03^*^(0.24)	0.49 (0.01)				
**BPI severity**							
Active tDCS + MT	7.33 (7–9)	4.07(4–6)	7.14 (7–9)	0.02^*^	0.03^*^	0.77	0.46
Sham tDCS + MT	6.49 (5–8)	4.98(4–7)	6.23 (6–9)	0.03^*^	0.01^*^	0.94	0.23
Between-group effects*P*-value (δ)	0.43 (0.01)	0.04^*^(0.12)	0.22 (0.03)				
**SF-36**							
Active tDCS + MT	47.07 (38–52)	59.11(53–62)	48.18 (41–54)	0.04^*^	0.04^*^	0.14	0.68
Sham tDCS + MT	44.11 (30–48)	51.81(48–58)	43.48 (38–47)	0.03^*^	0.01^*^	0.19	0.42
Between-group effects*P*-value (δ)	0.37 (0.09)	0.01^*^(0.72)	0.12 (0.09)				

IQR, Interquartile range. MPQ, McGill Pain Questionnaire. BPI, Brief Pain Inventory. SF-36, Medical Outcomes 36-item short- form health survey questionnaire.

* p < 0.05 (Within Groups and Between Groups). W, Kendall's test. δ = Cliff's delta. Effect size (Small = 0.1, Medium = 0.3, Large = 0.5).

### Secondary Outcomes

#### Pain Severity and Interference

Results from the ANOVAs examining session effects for BPI scale are shown in Table 2. The scores of Pain Severity Index were statistically similar at the baseline, but a significant difference was seen between the groups after the intervention, with the active tDCS+MT group having a lower mean score. Moreover, this change was not stable at the 3-month follow-up. For pain severity, during the trial there was a progressive mean fall in Pain Interference Index from baseline for both groups. However, the active tDCS+MT had better improvement than the sham tDCS+MT group at the endpoint. There was no maintenance of treatment effect, either active or sham group at follow-up (Figure 4).

#### Quality of Life

The quality of life post-treatment was significantly increased for both groups at the endpoint. However, comparisons between the baseline and the 3-month evaluation showed an improvement in SF-36 scores only in the active tDCS+MT group (Table 2). Figure 4 shows that the results appeared to follow the patterns of MPQ and BPI, whereby the change in scores seemed to be not maintained over time, i.e., there is a significant difference between the groups only during treatment, in favor of the active tDCS+MT.

### Effect of Treatment On Primary and Secondary Outcomes Considering Covariates

We investigated how group factors and covariates are associated with primary and secondary outcomes, using univariate linear regression analyses as parameter estimates (Table 3). As can be seen, belonging to the active tDCS + MT group was associated with an increased change in pain relief. Baseline anxiety was negatively associated with changes in the MPQ score. In addition, VAS adjusted index correlated negatively with changes in quality of life for both groups. No significant differences were observed in other clinical and demographic characteristics (*p* < 0.05).

**Table 3:** Univariate linear regression models for the effects of treatment groups (Active and Sham-tDCS + MT), anxiety (as a Covariate) and the interaction treatment^*^baseline pain (VAS index) on deltas of outcome measures.

**Dependent variable**	**B**	**SEM**	***F***	***P***
**Δ** **MPQ**				
Intercept	1.19	27.29	0.06	0.842
Active group	71.29	33.14	2.34	**0.034**
Sham group^a^	.	.	.	.
IDATE-S	−2.40	1.08	−2.16	**0.019**
Active group* VAS score	−1.85	0.82	−2.91	**0.041**
Sham group* VAS score	0.62	0.47	1,17	0.310
**Δ** **BPI–Interference**				
Intercept	−8.29	15.46	−0.41	0.567
Active group	37.18	13.34	1.38	0.173
Sham group^a^	.	.	.	.
IDATE-S	−1.42	0.95	−1.23	0.157
Active group* VAS score	−0.92	0.53	−1.33	0.196
Sham group* VAS score	−0.39	0.22	−0.11	0.812
**Δ** **BPI–Severity**				
Intercept	−6.37	14.05	−0.38	0.426
Active group	31.29	9.76	1.42	0.718
Sham group^a^	.	.	.	.
IDATE-S	−1.51	0.82	−1.49	0.276
Active group* VAS score	−0.89	0.47	−1.51	0.179
Sham group* VAS score	−0.47	0.31	−0.19	0.724
**Δ** **SF-36**				
Intercept	75.11	23.35	2.98	**0.002**
Active group	−49.02	34.26	−1.37	0.152
Sham group^a^	.	.	.	.
IDATE-S	−1.39	0.82	−1.28	0.178
Active group* VAS score	−2.68	0.83	−3.37	**0.001**
Sham group* VAS score	−2.46	0.77	−3.19	**0.001**

*SEM, standard error of the mean*.

a *Comparative group, to which values are referenced to. Significance level was P < 0.05. VAS, Visual Analog Scale. MPQ, McGill Pain Questionnaire. BPI, Brief Pain Inventory. SF-36, Medical Outcomes 36-item short-form health survey questionnaire. Bold text indicates a statistically significant difference with p < 0.05*.

## Discussion

The present double-blind, sham-controlled pilot study provides evidence that tDCS combined with mirror therapy promotes analgesia in multidimensional aspects, reducing the severity and interference of pain with daily activities, as well as improving quality of life levels in patients with neuropathic pain. Interestingly, the effects of active tDCS on primary and secondary outcomes were partially dependent on baseline levels of pain and anxiety. However, these benefits were not maintained in the long-term (3-month follow-up). As far as we know, this is the first study to assess the effects of tDCS paired with physical therapy in patients with brachial plexus injury.

Our results are similar to previous studies that found synergistic effects of tDCS and mirror therapy in pain relief and increased quality of life in other clinical conditions (12, 39, 40). Recent studies have shown that tDCS application during mirror therapy promotes greater facilitation of corticospinal excitability and consequently enhances the effects of neuronal reorganization promoted by physical therapy (41, 42). In fact, in brachial plexus injury, there is an imbalance between the excitatory and inhibitory circuits, with a distortion in body perception related to the alteration of cortical maps (43). Although the mechanisms of action of synergism between tDCS and mirror therapy are not yet well-established, it is suggested that observation of movements leads to a reduction in intracortical inhibition (44) and that tDCS causes modulation of upward nociceptive pathways (45, 46). It should also be noted that an association of the two therapies could modulate the impaired information in these circuits, changing the synaptic transmission (19). However, further studies are needed to elucidate such questions.

When considering the interaction between treatment and the pain and anxiety levels at baseline, it becomes clear that these variables had important effects in the group that received active tDCS and mirror therapy. It has been indicated that both the pain and anxiety levels are variables that modify treatment efficacy in patients with chronic pain (40). Similarly, higher levels of pain and anxiety have been linked to higher disability scores in neuropathic pain (5, 47). Therefore, especially for this population and perhaps for other conditions with similar chronic pain, patients with high pain and anxiety levels may not benefit from treatment with tDCS and mirror therapy, leading to small changes in performance tests.

We also found that treatment effects were dependent on the pain level at baseline in relation to the quality of life. The difference here is that, unlike the primary outcome measured by the McGill questionnaire, this association was not only present for the active group, whose tests presented a larger effect size. Although it is not clear why this relationship was also observed in the sham group, when we consider these associations, it should be noted that patients in this group also received an intervention arm of mirror therapy. Thus, for the quality of life construct (which has different factors from those that compose the pain component), how much pain the patient has before starting treatment should probably also be considered when applying mirror therapy, regardless of whether it is associated with neurostimulation.

Our results did not show benefits in pain and quality of life being maintained at follow-up for both groups. We can speculate that the duration of therapy we employed (12 sessions, with 30 min per session) may not be sufficient to induce significant long- term retention effects in patients with neuropathic pain. Studies with other populations show lasting visual illusion benefits in reducing pain (48), suggesting potential for conducting clinical trials to assess whether multiple sessions or longer sessions could alter cortical plasticity, leading to long-term analgesia in patients with brachial plexopathy.

Although the present study may bring important considerations for non-invasive treatment in patients with neuropathic pain, these findings should be viewed with caution for some reasons. For example, in the present work, we did not employ neuroimaging or computational modeling techniques to control cortical changes. In order to minimize this bias, we controlled the eligibility criteria, performed randomization among the groups and, used statistical inferences to weigh possible moderations of variables associated with response prediction. In a complementary way, it is worth mentioning that these inferences are also limited due to the lack of a healthy control group and a placebo mirror therapy protocol. In addition, we had a predominantly male sample, which limits our conclusions to this gender, although it should be noted that brachial plexus avulsion has a higher prevalence in males (49, 50). Small sample size is a limitation of our study, and further larger and multi-center trials are needed to fully assess the role of these interventions in the clinical management of neuropathic pain after brachial plexus injury.

Overall, our findings highlight two important conclusions. First, 12 sessions of anodic tDCS on the primary motor cortex combined with mirror therapy has a superior effect in promoting analgesia and improving quality of life compared to simulated stimulation and mirror therapy. The pain and anxiety levels before treatment had a relevant effect on our model when we considered the disability generated by neuropathic pain measured by the outcomes of the active group. These findings encourage developing studies involving multimodal therapies in other types of chronic pain and considering possible effect modifying variables.

## Data Availability Statement

The raw data supporting the conclusions of this article will be made available by the authors, without undue reservation.

## Ethics Statement

The studies involving human participants were reviewed and approved by Research Ethics Committee–Health Science Center–Federal University of Paraíba (CEP-CCS-UFPB). The patients/participants provided their written informed consent to participate in this study.

## Author Contributions

EB, CF, CC, and RG: study conception and design. CF, CC, RG, and EB: data collection and analysis. SA, CM, CC, and RG: writing and/or critically revising the manuscript. All authors contributed to the article and approved the submitted version.

## Conflict of Interest

The authors declare that the research was conducted in the absence of any commercial or financial relationships that could be construed as a potential conflict of interest.
